# Development and psychometric validation of the frontline health workers’ occupational risk and characteristics in emergencies index (FORCE-index) – The covid Hospital cohort study

**DOI:** 10.1016/j.puhip.2025.100582

**Published:** 2025-01-10

**Authors:** Synne Øien Stensland, Kristina Bondjers, John-Anker Zwart, Leiv Arne Rosseland, Dan Atar, Jan Olav Christensen, Dagfinn Matre, Kristin Alve Glad, Tore Wentzel-Larsen, Hilde Wøien, Grete Dyb

**Affiliations:** a Norwegian Centre for Violence and Traumatic Stress Studies, Oslo, Norway; b Department of Research and Innovation, Division of Clinical Neuroscience, Oslo University Hospital, Oslo, Norway; c Institute of Clinical Medicine, University of Oslo, Norway; d Department of Research and Development, Division of Emergencies and Critical Care, Oslo University Hospital, Oslo, Norway; e Division of Cardiology, Oslo University Hospital Ulleval, Oslo, Norway; f National Institute of Occupational Health, Norway; g Centre for Child and Adolescent Mental Health, Eastern and Southern Norway, Oslo, Norway; h Division of Emergencies and Critical Care, Oslo University Hospital, Oslo, Norway; i Unit for Health Scientific pedagogics, Faculty of Medicine, University of Oslo, Oslo, Norway

**Keywords:** Covid-19, Health emergency, Infectious outbreak, Health response, Preparedness, Preparedness planning, Health Personnel, Health care worker, Frontline, Hospital, Occupational exposure, Work environment, Occupational Health, Occupational diseases, Occupational stress, Job characteristics, Stress, Psychometric, Index, Scale

## Abstract

**Objectives:**

A lack of tools for the systematic identification of frontline health workers' changing occupational risks, characteristics, and needs, poses a major barrier to supporting vital personnel to stay in practice through health emergencies and beyond. The current study reports on the development and psychometric evaluation of the Frontline health workers’ Occupational Risk and Characteristics in Emergencies index (FORCE-index).

**Study design:**

The Covid hospital study is a large, multisite, four-wave, open cohort study of frontline health workers responding to the first four waves of the COVID-19 pandemic (2020–2022).

**Methods:**

2496 frontline health workers responded to questionnaires assessing various aspects of their work environment. Using exploratory factor analysis, we estimated the latent structure of the FORCE-index at the first and second waves. This structure was evaluated using confirmatory factor analysis at the third and fourth waves. The internal consistency of the instrument's subscales (e.g., factors) was evaluated using omega reliability, Cronbach's alpha coefficient, and mean inter-item correlation.

**Results:**

A nine-factor solution provided best fit to the data. These factors mapped onto the following aspects of the work environment; competency, stress management, familiarity, workload manageability, work performance, infection safety, personal protective equipment, social safety, and social support. Internal consistency for the full FORCE-index and the nine factors was satisfactory.

**Conclusions:**

The initial psychometric validation indicates that the FORCE-index is a valid measure which can be used by health authorities, services, and institutions to adequately and systematically assess central aspects of frontline health workers’ work environment that are commonly challenged in health emergencies.

## 1 Introduction

A healthy and productive health service workforce is the most vital resource in an effective response to severe disease outbreaks (e.g., epidemics and pandemics) and other health emergencies [1,2]. Yet, frontline health workers run a heightened risk of severely adverse health outcomes, including hospitalization and death due to infectious disease. Up to one third experience stress-related health problems, such as anxiety, depression, posttraumatic stress, or burnout [[3], [4]]. Hospitals constitute particularly high-risk work environments during severe infectious disease outbreaks [1,3], where frontline health workers in direct contact with patients with confirmed or suspected disease commonly run the highest risk of adverse health outcomes [1,3].

Even before the Coronavirus Disease 2019 (COVID-19) pandemic international authorities repeatedly urged health institutions and services to develop need-based interventions efficiently supporting frontline health workers to stay in practice through health emergencies and beyond [5]. Yet, interventions targeting the work-related needs of frontline health workers remain largely underdeveloped and unimplemented [1,3,6]. Frameworks like Emergency Responder Health Monitoring and Surveillance (ERHMS) [7] and Occupational Health and Safety Management Systems [9], as well as the Healthcare Worker Exposure Response & Outcomes (HERO) Registry [8] all recommend identifying and monitoring work-related characteristics, tasks, and challenges faced by frontline healthcare workers during crises. For frontline workers responding to health emergencies, work environmental factors seem to play an important role in self-efficacy, resiliency, productivity, and health. The most important aspects of the work environment relate to workplace preparedness; facilitating fulfillment of job tasks and work stress management; unpredictability in work environment and teams; high workload and performance; protection and safety; and access to a supportive learning environment [3,10,11]. Although the importance of the work environment for the health, well-being, and productivity of personnel is well established [1,[12], [13], [14]], the abovementioned aspects of the work environment, commonly challenged in health emergencies, are largely unaccounted for in most work environmental measures. Recent efforts to address this gap, include the Health Care Workers' Concerns in Infectious Outbreaks Scale (HCWCIOS). The scale was developed using data from a convenient sample of 354 Iranian health care workers, and identified six key factors, including inadequate preparedness, lack of knowledge, risk perception, affected social relations, work pressure, and absenteeism [15].

Recent findings from systematic reviews pinpoint the current lack of valid and systematic identification of frontline health workers' multifaceted and changing occupational risks, characteristics, and needs during health emergencies as a major barrier to developing effective support over time [1,3,16,17]. To meet this demand, the current study describes the development and psychometric evaluation of a new, comprehensive measurement tool for assessing Frontline health workers’ Occupational Risk and Characteristics in Emergencies, the FORCE-index. We use data from a multisite hospital-based study, including 2496 frontline health workers from four central, public, university hospitals. Data collections were timed to capture the infection waves from the initial outbreak through to the fourth wave, allowing us to measure the multi-faceted occupational characteristics, tasks and challenges that influence hospital frontline workers' ability to maintain effective practice over time, whilst responding to an evolving health crisis. The FORCE index builds upon existing frameworks and instruments, as well as specifically addressing the unique and prolonged challenges faced by healthcare workers during extended health crises. By focusing on both immediate and long-term aspects of health emergency response, the FORCE index seeks to fill a critical gap in our understanding and measurement of the psychosocial work environment in healthcare during crises.

The specific objectives of the current study were.

• To develop a comprehensive tool (FORCE-index) for assessing frontline health workers' occupational risks and characteristics during health emergencies.
• To evaluate the psychometric properties of the FORCE-index, including its factor structure and internal consistency.
• To determine the reliability and structural validity of the FORCE-index across multiple waves of the COVID-19 pandemic.

## 2 Methods

### 2.1 Study Design

**Fig. 1:**
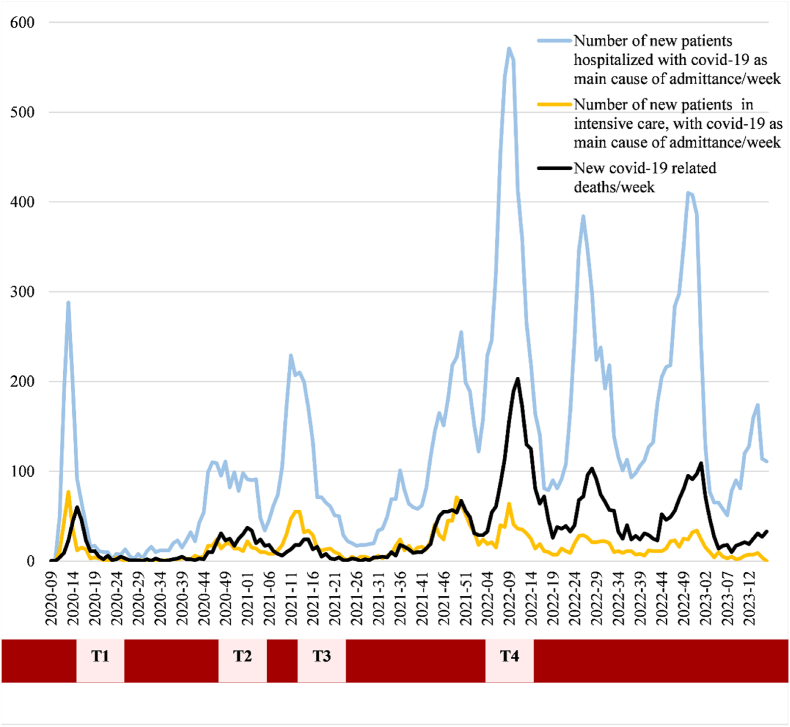
Data collection timepoints 1, 2, 3 and 4 (T1-T4, year - week) in the Covid Hospital Study in relation to national numbers of hospitalized patients, patients in intensive care and deceased during the four primary waves of the COVID-19 pandemic in Norway (Data from the Norwegian national emergency preparedness registry for COVID-19, the Norwegian Institute of Public Health).

The Covid Hospital Study is a prospective, open-cohort, multi-center study of hospital frontline workers conducted during the four main waves of the COVID-19 pandemic (2020–2022) (Fig. 1). The scale development was based on the guidelines described in Scale Development: Theory and Applications [18] and the Consensus-based Standards for the Selection of Health Status Measurement Instruments checklist [19].

### 2.2 Setting and participants

Hospital frontline workers, working directly or indirectly with patients with suspected or diagnosed COVID-19, were recruited from four large-scale public, university hospitals across Norway, from the south-eastern part to the far north in Tromsø, to provide a comprehensive view of healthcare worker experiences in varied environments, including: Oslo University Hospital (OUH), Akershus University Hospital (Ahus), St. Olav's University Hospital (St. Olavs) and the University Hospital of Northern-Norway (UNN). These hospitals were selected as these admitted the vast majority of severely ill patients in their respective regions, reflecting great variation in infection rates, sociodemographic and logistical challenges. The study was designed as an in vivo, open cohort, prospective, web-based study, with data collection conducted during major infection waves. More specifically, data collection time points were strategically planned to be aligned with peaks in infection rates to specifically capture the occupational characteristics, tasks, challenges and consequences experienced by health care workers responding to an evolving health emergency. Thus, frontline workers at the participating hospitals were invited to take part in data collections in April–June 2020 (T1), November–December 2020 (T2), April–May 2021 (T3), and January–February 2022 (T4). Invitations to participate were sent out to all eligible participants using the hospitals' normal channels of communication with their staff (e.g., e-mail, SMS, and online bulletin boards). General information about the study was spread on-site at each hospital (including the intranet), as part of the local recruitment process. Consenting participants logged in using their social security number and were then administered the questionnaire. Participants were mainly nurses, physicians, or other hospital personnel (e.g., physiotherapists, nutritionists, assistant nurses, ambulance personnel, and psychologists).

#### 2.2.1 Ethical Considerations

Invited hospital frontline personnel were provided with written information explaining the purpose and methods of the study, including personal and data protection aspects, to ensure voluntary, informed consent to participation. The study received ethical approval from the Norwegian Ethical Review Authority (#130944).

#### 2.2.2 Role of the funding source

The study was funded by the Research Council of Norway (#312750/H40) and the Norwegian Directorate of Health (#14/1587-184). The study sponsors were not involved in the study design, data collection, analysis, or interpretation, in writing the report, or in the decision to submit the paper for publication.

### 2.3 Measures

Participants answered a questionnaire consisting of items assessing demographic data, professional role, pandemic exposure, as well a set of items measuring frontline workers’ job characteristics and tasks, referred to as the FORCE-index.

#### 2.3.1 Participants’ demographics, professional characteristics and pandemic exposure

Participants' age and sex were determined using their social security numbers. Participants' profession was categorized as ‘nurse’, ‘physician’, or ‘other frontline worker’, the latter encompassing all other hospital personnel. Additionally, the participants reported on years of clinical experience and hospital adherence. Direct contact with patients with suspected or diagnosed COVID-19 on intermediary/intensive care units or ordinary wards/covid cohorts during the past month was categorized as ‘Direct contact, but not with the severely ill’ and ‘Direct contact with the severely ill’, respectively. For personnel working at the hospitals during the outbreak who reported no direct contact with patients with suspected or diagnosed COVID-19, level of exposure was categorized as ‘Indirect contact’, in line with recommendations by Pollock et al. (2020) [1].

#### 2.3.2 The frontline health workers’ occupational risk and characteristics in emergencies index (FORCE-index)

To develop the FORCE-index, we aimed to identify critical organizational and psychosocial work factors that support resiliency and health among frontline workers during health crises. Our multidisciplinary project group, heading the development of the items, included experts from various fields, including occupational health, emergency medicine, psychiatry/psychology, paediatrics, neurology, clinical medicine, nursing, and hospital leadership, ensuring a comprehensive understanding of the specific work environment challenges. The initial item pool was generated based on theoretical frameworks and empirical knowledge. More specifically, the project group thoroughly reviewed existing empirical studies and theoretical evaluations related to occupational health, stress management, and emergency medicine [1,3,10,[12], [13], [14],^16^], and consulted frontline workers involved in the hospital COVID-19 response, through a user panel of the study, to ensure development of items grounded in real-world experiences. To establish face validity, we conducted qualitative assessments where both the user panel and project group reviewed each item for meaningfulness, relevance, clarity, comprehensiveness, user-friendliness, and feasibility. This led to modifications, whereof some questionnaire items were deleted or divided while new items were added leaving us with a total of 32 items at T1 and 53 largely overlapping items at T2 (Supplementary Tables 3 and 5) through to T4. All items were transformed to fit a 0–10 scale, and appropriate items were reversed to ensure coherence of the scale (0 reflecting more negative responses/experiences, compared to 10 reflecting more positive responses/experiences).

### 2.4 Statistical analyses

Our approach to developing the FORCE index items in the initial phase was primarily qualitative, driven by the urgent need to assess frontline workers' experiences during the rapidly evolving COVID-19 pandemic. Descriptive analyses were conducted for comparison of participants across the four time-points (i.e., T1-T4). Further, participants with and without missing values for the FORCE-index items at T1 (pilot) and T2 were compared in regard to background factors and pandemic exposure.

To evaluate the psychometric properties of the FORCE-index, we conducted Exploratory Factor Analyses (EFAs) to identify underlying factors related to frontline workers' job characteristics (Supplementary Tables 1–5). The EFAs, using only complete cases, were conducted at T1 (pilot) and T2. The number of factors retained in the EFAs was determined based on visual inspection using scree plots in light of theoretical assumptions, convergence and parsimony across the two time points. Due to assumption of correlation among factors, principal axis factoring analyses (PAF) with promax rotation were employed [[18], [20]] once factorability was confirmed. Sampling adequacy for data factorability was determined by a Kaiser-Mayer-Olkin (KMO) value greater than 0·50, and significance of Bartlett's test of sphericity (*p* < 0·05). Based on an assessment of results across the two EFAs, factors with salient coefficients interpreted as substantively meaningful, were retained as items in the final scale. Items with factor loadings <0·5 in absolute value were excluded, as were complex loadings that were salient on more than one factor, to honor the concept of simple structure [18]. Each extracted factor was given a meaningful name.

Finally, results from the EFAs were reassessed while accounting for a priori empirical and theoretical knowledge to determine distinct first and second order factor models to be tested in separate Confirmatory Factor Analyses (CFAs) at T3 and T4 [1,3,10,[12], [13], [14],^16^].

For the CFAs, the mean and variance adjusted weighted least squares (WLSMV) estimator was used to estimate the factor loadings, as it provides accurate standard error for ordinal level indicators. Model fit was evaluated using the following measures of fit: a non-significant chi-square result indicates good model fit; Comparative Fit Index (CFI) and Tucker-Lewis Index (TLI) values ≥ ·90 and ≥ ·95 indicating adequate and excellent fit, respectively; and Root Mean Square error of Approximation (RMSEA) values ≤·08 and ≤·06 indicate adequate and excellent fit, respectively [[21], [22], [23]]. To compare proposed alternative models, we relied on changes (Δ) in the RMSEA result, as this index includes penalties for model complexity, and Δ ≥ ·015 is indicative of significant changes in the fit of the respective models [24]. The ratio of chi-squares with respect to the degrees of freedom (χ2/df < 2·00) was also evaluated, with values < 2 indicating superior fit. Internal consistency reliability of the potential subscale (range from 0 to 10) was evaluated using omega reliability, Cronbach's alpha coefficient, and mean inter-item correlation. IBM SPSS (version 28·0, SPSS Inc., Chicago, IL, USA) and R version 4·2·2, packages lavaan [25], semTools [26], and psych [27] were used for the analyses.

Confirmatory Factor Analyses (CFAs) were subsequently performed at T3 and T4 to validate the scale structure developed from earlier analyses (Table 2 and Supplementary Table 6). Internal consistency and reliability was assessed using omega reliability coefficients and Cronbach's alpha (Table 3).

**Table 1:** Demographics, professional role, clinical experience, and pandemic exposure among the 2496 hospital personnel participating at one or more time-points corresponding in time with the four main waves of the COVID-19 pandemic (2020–2022).

			T1	T2	T3	T4
			N = 687	N = 1088	N = 831	N = 978
			N (%)/mean (SD)	N (%)/mean (SD)	N (%)/mean (SD)	N (%)/mean (SD)
**Demographics**						
	Sex, female		548 (79·8)	820 (75·4)	582 (70·0)	735 (75·2)
	Age		43·5 (10·9)	42·4 (11·6)	44·4 (11·7)	45·1 (11·8)
**Profession, experience, and workplace**						
	Profession					
		Nurse	410 (59·7)	485 (44·6)	385 (46·3)	437 (44·7)
		Physician	145 (21·1)	192 (17·6)	151 (18·2)	186 (19·0)
		Other hospital personnel	132 (19·2)	411 (37·8)	294 (35·4)	354 (36·2)
	Working clinically (with patients)			924 (85·4)	702 (84·8)	819 (84·5)
		Clinical experience, years	16.6 (10·2)	14.7 (10·3)	16.0 (10·6)	16.7 (11·1)
	Workplace					
		South-East: OUS/Ahus	620 (90·6)	699 (64·2)	563 (67·9)	708 (72·7)
		Mid/North: St.Olav/UNN	64 (9·4)	389 (35·8)	266 (32·1)	266 (27·3)
**Pandemic exposure**						
	Current contact with suspected/diagnosed COVID-19 patients					
		Direct contact with the severely ill	276 (43·1)	230 (21 ·2)	274 (33·0)	252 (25·8)
		Direct contact, but not with the severely ill	166 (24·2)	225 (20·8)	117 (14·1)	251 (25·7)
		Indirect or potential contact	199 (31·0)	629 (58·0)	440 (52·9)	473 (48·5)

**Table 2:** Model fit statistics for the first and second order models at T3 and T4.

	c [2]	*df*	CFI	TLI	RMSEA (95 % CI)			SRMR
First order model T3	591·747∗∗∗	341	·980	·976	·031	(·027	.·035)	·049
Second order model T3^a^	959·86∗∗∗	362	·952	·946	·046	(·043	·050)	·063
First order model T4	586·906∗∗∗	341	·980	·976	·029	(·025	·033)	·046
Second order model T4^a^	1134·441∗∗∗	362	·937	·929	·049	(·046	·053)	·063

*Note.* χ^2^ = chi-square; *df* = degrees of freedom; CFI = comparative fit index; TLI = Tucker-Lewis index; RMSEA = root mean square error of approximation.*∗∗∗p* < ·0001.

**Table 3:** Internal consistencies of the full FORCE-index and nine factors.

	T3			T4		
	Alpha	CR	MIIC	Alpha	CR	MIIC
Competency	·88	·88	·69	·86	·86	·67
Stress management	·94	·94	·84	·94	·95	·85
Familiarity	·67	·68	·41	·74	·74	·49
Workload manageability	·82	·82	·54	·82	·81	·52
Work performance	·85	·84	·65	·83	·82	·62
Infection safety	·81	·82	·67	·75	·76	·59
Personal protective equipment	·80	·81	·40	·78	·78	·36
Social safety	·65	·56	·37	·61	·57	·32
Social support	·84	·86	·72	·80	·83	·66

*Note.* Alpha = Cronbach's alpha; *CR*= Composite reliability; MIIC = mean inter-item correlation.

## 3 Results

### 3.1 Participant characteristics

Altogether, 2496 frontline workers participated at one or more of the four data-collection time-points, whereof N = 687 participated at T1, N = 1088 at T2, N = 831 at T3, and N = 978 at T4 (Table 1). About one third participated more than once (N = 766, 30·7 %), 513 (20·6 %) participated twice, 185 (7·4 %) three times, and 68 (2·7 %) four times. In total, 75·1 % (N = 1875) of participants were females. Mean age ranged from 42·4 (SD 11·6) – 45·1 (SD 11·8) years across the four waves, with the youngest attendee being 18 and the oldest 81 years of age. The majority of the participants had long clinical experience, were nurses or physicians, and worked in direct contact with patients with suspected or diagnosed COVID-19.

Frontline workers included in the EFAs at T1 (pilot) and T2, were significantly younger, more likely to be nurses or physicians (as opposed to other frontline workers), directly exposed, and have slightly shorter clinical experience, as compared to participants not included due to missing values on the FORCE-index (Supplementary Table 1).

Results from Bartletts test of sphericity and Kaiser-Mayer-Olkin (KMO) performed with data from the pilot (T1) and T2 indicated that data were factorable (Supplementary Table 2). As part of the initial process, reviewing developed items’ face relevancy and validity for inclusion versus exclusion in the FORCE index, 10 items from the T1 (Pilot) and 24 items from T2 were excluded, based on review of results of initial exploratory factor analyses (loading <0·5), incoherence with theory or lack of face validity (Supplementary Tables 3 and 5). Results from the EFAs including the remaining 22 items at T1 and 29 items at T2 demonstrated a cumulative explained variance (Extraction sums of squared loadings) of 55 % at T1 and 46 % at T2, and satisfactory factor loadings of the final items retained on the nine proposed factors; i) competency, ii) stress management, iii) familiarity, iv) workload manageability, v) work performance, vi) infection safety, vii) personal protective equipment (PPE) viii) social safety, and ix) social support (Supplementary Tables 3, 4a and 4b).

Based on these results, two competing models were proposed, tested for model fit, and compared using CFAs on data from T3 and T4. These models included one first order model (comprising the nine identified factors) and a second order model (including four overarching factors onto which the nine subfactors loaded) (Table 2 and Supplementary Table 6). The four overarching second order factors were termed: i) *preparedness*, encompassing competency and stress management, ii) *workday manageability*, encompassing familiarity, workload manageability, and work performance, iii) *hazard protection*, encompassing infection safety and personal protective equipment (PPE), and v) *social environment*, encompassing social safety and social support.

The first order model provided satisfactory model fit at both T3 and T4 (Table 2). The second order model provided slightly worse fit at both time points, and the covariance matrices for these models were not positive definite. Thus, the second order model was rejected in favour of the first order model. Factor loadings for the models are provided in the supplement (Supplementary Table 6).

Internal consistencies of the full FORCE-index and nine factors were considered acceptable for most subscales (Table 3). Alpha and composite reliability for the *familiarity* and *social safety* subscales were lower than desired, indicating a lower internal consistency for these two subscales.

## 4 Discussion

In this study, we describe the development and initial psychometric evaluation of the FORCE-index. The initial psychometric validation indicates that the FORCE-index is a valid measure which can be used by health authorities, services, and institutions to adequately and systematically assess the occupational characteristics and tasks frontline health workers experience when responding to an infectious disease outbreak.

The FORCE-index was developed and psychometrically tested using a comprehensive dataset, following frontline workers as they responded to the four main waves of the COVID-19 pandemic. As recommended, the initial items were developed, piloted, and revised based on theoretical and empirical knowledge of aspects of the work environment which impact the health and resiliency of personnel [1,3,14,28]. Results of the subsequent psychometric testing of the final 29 items provide support for the construct validity nine latent variables of the FORCE-index, largely corresponding to theoretical and empirical knowledge on work environment and including *competency*, *stress management, familiarity, workload manageability, work performance, infection safety, personal protective equipment, social safety* and *social support*. These factors seem to largely overlap with the factors identified by Yarahmadi et al., in their development of the Health Care Workers' Concerns in Infectious Outbreaks Scale (HCWCIOS), including *lack of knowledge* and *inadequate preparedness, work pressure, risk perception*, and *affected social relations* [15]. This similarity corroborates that the identified factors are salient work environmental aspects for healthcare workers during crises across diverse settings. However, the FORCE-index extends beyond the HCWCIOS demonstrating the stability of these factors over time, in a larger and more diverse sample. Also, the focus of the FORCE-index on occupational factors strengthening resiliency, may help guide organizations in their efforts to develop adequate interventions.

In this study, results from the CFAs provide support for the construct validity of the FORCE-index, indicating the best fit for the first order nine-factor model as compared to a proposed theoretically driven second order model (grouping factors in the four proposed overarching factors of preparedness, workday manageability, protection, and social environment). A closer look at the challenges evolving across the four main waves of the pandemic may help explain this finding. During the early phase of the COVID-19 pandemic (waves 1 and 2), response primarily required handling the upsurge of severely ill patients with a formerly unknown virus, despite a lack of knowledge of specific transmission patterns, effective protection, practices, treatments [29]. Sweeping reorganizations of the overall structure of services and procedures were undertaken, including reallocating personnel, demanding a high level of flexibility among the frontline health workers in the midst of the uncertainty and unpredictability of changing working teams, chains of command, schedules, workload, and tasks. Worldwide mortality and morbidity among frontline workers were high, and many experienced the loss of patients, colleagues, friends, and family [[30], [31], [32]]. Adequate PPE was in short supply, and personnel were commonly asked to limit their use, or were offered inadequate equipment, potentially jeopardizing the health of personnel and patients or preventing the provision of effective care. Frontline workers frequently reported significant stress related to health problems and inadequate personal protection [4]. In the later phase (waves 3 and 4), there was increased knowledge about the virus, improved treatment and practices, access to vaccinations, more effective and available PPE, and better routines for reallocation of personnel, all of which helped to improve preparedness, treatment outcomes, predictability, and safety. However, in this later phase, demand for hospitalization, due to the easing of public protection measures, commonly overwhelmed service capacity, leading to a calculated upsurge in severely ill patients alongside increased sick leave among personnel. This retrospective overview of frontline workers' occupational challenges during the different COVID-19 waves may, in part, strengthen confidence in the validity of the first order model, as the identified factors seem to interrelate somewhat differently across the four waves and in regard to frontline health workers’ level of risk.

The severe work environmental risks related to health emergency response add to well-known problems in the health sector, which already has the highest estimated prevalence of work-related stress [1,3,10,33,34]. For decades, work environmental research has advanced knowledge of the importance of job characteristics for health, resiliency, and stress [12]. Prior research has shown that high physical and psychological demand, in combination with low control or *decision* latitude, low social support and high *job insecurity*, pose particularly important interrelated aspects of the work environment predicting risk of chronic stress and adverse health outcomes among nurses, physicians, and other professions alike [1,[12], [13], [14],^17^]. The factors identified in the current study build on and relate to these acknowledged constructs, while integrating work environmental interrelated aspects challenging frontline health workers responding to health emergencies more specifically.

The challenges encountered by frontline health workers during infectious disease outbreaks and other health emergencies may, in part, reflect an exacerbation of common challenges in healthcare, largely unaccounted for in current research and practice [34,35]. Thus, it is reasonable to suggest that the FORCE-index could be useful in measuring job characteristics and tasks in a broader healthcare context.

### 4.1 Strength and limitations

The ‘in vivo’ cohort design of this large study with directly and indirectly exposed frontline personnel working in high-risk hospital environments throughout the four main waves of the COVID-19 pandemic is an empirical strength. While the nature of the study (e.g. following the urgency of the pandemic and collecting real-time data from study participants) prevented us from collecting data allowing for quantitative assessment of for example content validity, this was compensated by an iterative process of expert review and statistical analysis across multiple time points. This strengthened the questionnaire's validity and reliability. That said, variation in participation and inclusion in analyses over time may have led to some selection biases. In particular, directly exposed frontline workers were overrepresented in the complete case EFAs at T1 and T2, corresponding to COVID-19 waves 1 and 2. During these first two waves, prior to implementation of vaccinations, Norway was at emergency alert level 2, after which risk of exposure declined. The identified constructs could therefore be particularly applicable for more high-risk environments, such as intermediary and intensive care units. However, the results seem to apply beyond the highest risk settings, as the first order model provided satisfactory model fit at both T3 and T4, with about half of participants being indirectly exposed. Also, as the study included frontline workers with different professional backgrounds and levels of exposure, representative of most hospital settings and many health institutions alike, results are likely to be broadly applicable, although this remains to be examined. The instrument was developed and psychometrically tested to measure the job characteristics and tasks of frontline workers responding to a long-lasting infectious outbreak, with repeated infectious waves over time. There is a possibility that results may have been different in a setting with a more or less virulent virus, shorter duration of outbreak, or another type of health emergency. Of particular note this study was set in a high-income country setting. In a health emergency setting, efficiency of response highly depends on the number and operative functioning of trained individuals and teams over time, including their access to technological and organizational resources and flexibility [36]. Globally, the number of health care workers, their training and access to resources vary greatly, as do the challenges of the emergency exposed populations they serve [37]. Thus, it is highly likely that the occupational challenges, tasks and characteristics health care workers face during health emergencies may qualitatively differ relating to the setting. On the other hand, as discussed above, it is also possible that the FORCE-index may be applicable for measuring certain shared aspects relating to job characteristics and tasks of frontline workers in health care more generally, beyond infectious outbreaks and health emergencies. Thus, potentially, the FORCE-index might serve as a valuable tool beyond high income country settings. Nonetheless, to ensure generalisability of these findings, including the rejection of the theory driven second order model in this study, the FORCE-index would need to be psychometrically tested in other samples. More specifically we recommend comparative studies across various healthcare settings, including LMIC settings and regions to better understand context-specific stressors faced by health care workers responding to health emergencies. Additionally, further research will need to investigate how knowledge of specific occupational stressors and risks may help organizations develop effective support systems strengthening resilience among healthcare workers during crises over time.

Widespread adoption of common, open-access, validated tools within existing frameworks and practices, such as the ERHMS [7] and Occupational Health and Safety Management Systems [9], as well as the HERO Registry [8], could help strengthen evidence base, targeting and effectiveness of health authorities’ policy and decision-making processes and related resource allocation, to more effectively support frontline health workers responding to health emergencies across diverse settings [1]. Implementation of the FORCE-index within existing frameworks and practices, to enable systematic identification and monitoring of the multi-faceted occupational characteristics, tasks and challenges faced by frontline workers responding to health emergencies over time, could prove helpful.

## 5 Conclusions

The FORCE-index is a reliable and validated instrument for quantitatively assessing frontline workers’ job characteristics and tasks while responding to an infectious outbreak. Timely and repeated use of the FORCE-index could help services, organizations and authorities develop and adjust need-based organizational interventions, efficiently supporting frontline workers to stay in practice through health emergencies and beyond.

## Conflict of interest

The study received ethical approval from the Norwegian Ethical Review Authority (reference # 130944). The study was funded by the Research Council of Norway (project # 312750) and the 10.13039/501100014232Norwegian Directorate of Health (project # 14/1587-184). The study sponsors were not involved in the study design, data collection, analysis, or interpretation, in writing the report, or in the decision to submit the paper for publication.

The authors thank the participants in the Covid Hospital Study, and the collaborating partners; the National Institute of Occupational Health in Norway, the Akershus University hospital (Ahus), St. Olav's University hospital (St. Olav's), the University hospital of Northern-Norway (UNN), the Oslo University Hospital (OUH), the Norwegian Centre for Violence and Traumatic Stress Studies (NKVTS), the Regional resource centre for violence, traumatic stress and suicide prevention (East) and the Norwegian University of Science and Technology.

The authors report no conflicts of interest.

## References

[1] Pollock A., Campbell P., Cheyne J. (2020). Interventions to support the resilience and mental health of frontline health and social care professionals during and after a disease outbreak, epidemic or pandemic: a mixed methods systematic review. Cochrane Database Syst. Rev..

[2] Emanuel E.J., Persad G. (2023). The shared ethical framework to allocate scarce medical resources: a lesson from COVID-19. Lancet.

[3] Brooks S.K., Dunn R., Amlôt R., Rubin G.J., Greenberg N., Systematic A. (2018). Thematic review of social and occupational factors Associated with psychological outcomes in healthcare Employees during an infectious disease outbreak. J. Occup. Environ. Med..

[4] Muller A.E., Hafstad E.V., Himmels J.P.W. (2020). The mental health impact of the covid-19 pandemic on healthcare workers, and interventions to help them: a rapid systematic review. Psychiatry Res.

[5] World Health Organisation (2016).

[6] Tamminga S.J., Emal L.M., Boschman J.S. (2023). Individual-level interventions for reducing occupational stress in healthcare workers. Cochrane Database Syst. Rev..

[7] (2024). Emergency Responder Health Monitoring and Surveillance.

[8] HERO Healthcare Worker Exposure Response & Outcome.

[9] (2018). ISO 45001:2018(en) Occupational health and safety management systems - requirements with guidance for use. https://www.iso.org/obp/ui/en/#iso:std:iso:45001:ed-1:v1:en:ISO(theInternationalOrganizationforStandardization.

[10] Ruotsalainen J.H., Verbeek J.H., Mariné A., Serra C. (2015). Preventing occupational stress in healthcare workers. Cochrane Database Syst. Rev..

[11] van der Molen H.F., Nieuwenhuijsen K., Frings-Dresen M.H.W., de Groene G. (2020). Work-related psychosocial risk factors for stress-related mental disorders: an updated systematic review and meta-analysis. BMJ Open.

[12] Karasek R., Brisson C., Kawakami N., Houtman I., Bongers P., Amick B. (1998). The Job Content Questionnaire (JCQ): an instrument for internationally comparative assessments of psychosocial job characteristics. J. Occup. Health Psychol..

[13] Karasek R.A., Theorell T. (1990).

[14] Theorell T., Karasek R.A. (1996). Current issues relating to psychosocial job strain and cardiovascular disease research. J. Occup. Health Psychol..

[15] Yarahmadi S., Khademi M., Ebrahimzadeh F., Cheraghian T., Shahidi Delshad E. (2022). Development and psychometric properties of health care workers' concerns in infectious outbreaks scale. Front. Psychol..

[16] Aronsson G., Theorell T., Grape T. (2017). A systematic review including meta-analysis of work environment and burnout symptoms. BMC Publ. Health.

[17] Laurent A., Lheureux F., Genet M. (2020). Scales used to measure job stressors in intensive care Units: are they relevant and reliable? A systematic review. Front. Psychol..

[18] DeVellis R.F., Thorpe C.T. (2021).

[19] Mokkink L.B., Terwee C.B., Patrick D.L. (2010). The COSMIN checklist for assessing the methodological quality of studies on measurement properties of health status measurement instruments: an international Delphi study. Qual. Life Res..

[20] Watkins M.W. (2018). Exploratory factor analysis: a guide to best practice. J. Black Psychol..

[21] Bentler P.M. (1990). Comparative fit indexes in structural models. Psychol. Bull..

[22] Kline R.B. (2011).

[23] Steiger J.H. (1990). Structural model evaluation and modification: an interval estimation approach. Multivariate Behav. Res..

[24] Chen F., Curran P.J., Bollen K.A., Kirby J., Paxton P. (2008). An empirical evaluation of the Use of Fixed Cutoff points in RMSEA test statistic in structural Equation models. Sociol Methods Res.

[25] Rosseel Y. (2012). Lavaan: an R package for structural Equation modeling. J. Stat. Software.

[26] Jorgensen T.D., Pornprasertmanit S., Schoemann A.M., Rosseel T. (2022).

[27] Revelle W.R. (2017).

[28] Vangrieken K., De Cuyper N., De Witte H. (2023). Karasek's activation hypothesis: a longitudinal test of within‐person relationships. J. Organ. Behav..

[29] Chu D.K., Akl E.A., Duda S., Solo K., Yaacoub S., Schünemann H.J. (2020). Physical distancing, face masks, and eye protection to prevent person-to-person transmission of SARS-CoV-2 and COVID-19: a systematic review and meta-analysis. Lancet.

[30] Gholami M., Fawad I., Shadan S. (2023). The COVID-19 pandemic and health and care workers: findings from a systematic review and meta-analysis (2020-2021). Int J Public Health.

[31] Sahu A.K., Amrithanand V.T., Mathew R., Aggarwal P., Nayer J., Bhoi S. (2020). COVID-19 in health care workers - a systematic review and meta-analysis. Am. J. Emerg. Med..

[32] Mehta S., Machado F., Kwizera A. (2021). COVID-19: a heavy toll on health-care workers. Lancet Respir. Med..

[33] Meynaar I.A., Ottens T., Zegers M., van Mol M.M.C., van der Horst I.C.C. (2021). Burnout, resilience and work engagement among Dutch intensivists in the aftermath of the COVID-19 crisis: a nationwide survey. J. Crit. Care.

[34] Akram F., Pidcock M., Oake D., Sholler G.F., Farrar M.A., Kasparian N.A. (2023). "The usual challenges of work are all magnified": Australian paediatric health professionals' experiences during the COVID-19 pandemic. Int J Cardiol Congenit Heart Dis.

[35] Bondjers K., Lingaas I., Stensland S. (2023). "I've kept going" - a multisite repeated cross-sectional study of healthcare workers' pride in personal performance during the COVID-19 pandemic. BMC Health Serv. Res..

[36] Jakovljevic M., Timofeyev Y., Zhuravleva T. (2024). The impact of pandemic-driven care Redesign on hospital efficiency. Risk Manag Healthc Policy.

[37] Ahmed S., Chase L.E., Wagnild J. (2022). Community health workers and health equity in low- and middle-income countries: systematic review and recommendations for policy and practice. Int. J. Equity Health.

