# Supplementary material for: Development and psychometric validation of the frontline health workers’ occupational risk and characteristics in emergencies index (FORCE-index) – The covid Hospital cohort study
Source: Public Health Pract (Oxf). 2025 Jan 10;9:100582. doi: 10.1016/j.puhip.2025.100582 (PMC11787489; doi:10.1016/j.puhip.2025.100582)
Supplement: Multimedia component 1 [file mmc1.docx]

**Supplementary material**

| **Supplementary Table 1. Comparison between participants included in the explorative factor analyses, T1 (pilot) and T2.** | | | | | | | | | | | | | | |
| --- | --- | --- | --- | --- | --- | --- | --- | --- | --- | --- | --- | --- | --- | --- |
|  |  | | T1, N=687 | | | | |  | T2, N=1088 | | | | |  |
|  |  | | Included | |  | Not included | |  | Included | |  | Not included | |  |
|  |  | | N=366 | |  | N=321 | |  | N=390 | |  | N=698 | |  |
|  | | | Mean (SD)/N(%) | |  | Mean (SD)/N(%) | | p | Mean (SD)/N(%) | |  | Mean (SD)/N(%) | | p |
| Demographics | | |  |  |  |  |  |  |  |  |  |  |  |  |
|  | Age | | 42·09 | (10·46) |  | 45·19 | (1121) | <0·001^b^ | 39·50 | (10·64) |  | 44,07 | (11·75) | <0·001^b^ |
|  | Sex, female | | 297 | (81·1) |  | 251 | (78·2) | 0·336^a^ | 282 | (72·3) |  | 538 | (77·1) | 0·080^a^ |
| Profession | | | | |  |  |  | <0·001^a^ |  |  |  |  |  | <0·001^a^ |
|  | Nurse | | 244 | (66·7) |  | 166 | (51·7) |  | 224 | (57·4) |  | 261 | (37·4) |  |
|  | Physician | | 82 | (22·4) |  | 63 | (19·6) |  | 87 | (22·3) |  | 105 | (15·0) |  |
|  | Other | | 40 | (10·9) |  | 92 | (28·7) |  | 79 | (20·3) |  | 332 | (47·6) |  |
| Clinical experience, years | | | 15·32 | (9·60) |  | 17·95 | (10·74) | <0·001^b^ | 13,09 | (9·60) |  | 15·88 | (10·64) | <0·001^b^ |
| Pandemic exposure | | |  |  |  |  |  |  |  |  |  |  |  |  |
|  | Current contact with suspected/ diagnosed COVID-19 patients | |  |  |  |  |  | <0·001^a^ |  |  |  |  |  | <0·001^a^ |
|  |  | Indirect or potential contact | 47 | (13·5) |  | 152 | (51·9) |  | 134 | (34·4) |  | 495 | (71·3) |  |
|  |  | Direct contact, but not with the severely ill | 102 | (29·3) |  | 64 | (21·8) |  | 117 | (30·0) |  | 108 | (15·6) |  |
|  |  | Direct contact with the severely ill | 199 | (57·2) |  | 77 | (26·3) |  | 139 | (35·6) |  | 91 | (13·1) |  |
| PAF; principal axis factoring | | | | | | | | | | | | | | |

| **Supplementary Table 2. Results of the Principal Axis Factoring analyses, maximum likelihood (ML) method with promax rotation, at T1 (pilot) and T2.** | | | |
| --- | --- | --- | --- |
|  |  | T1 (pilot) | T2 |
| Bartletts test of sphericity | |  | Bartletts test of sphericity |
|  | Approximate chi square | 4766·098 | 8076·561 |
|  | df | 435 | 1081 |
|  | Sig. | <0·001 | <0·001 |
| Kaiser-Mayer-Olkin Measure of Sampling Adequacy(KMO) | | 0·885 | 0·859 |

| **Supplementary Table 3**. Presentation of the Frontline health workers’ Occupational Risk and Characteristics in Emergencies index (FORCE-index) items and factor loadings based on results from Explorative factor analyses: Principal Axis Factoring, Promax rotation, T1 (Pilot) - T2. | | | | | |
| --- | --- | --- | --- | --- | --- |
| **Item #** | | **Questionnaire items** | | **Factor loadings, PAF** | |
|  |  | **T1** | **T2** | **T1** | **T2** |
|  |  | **The 22-item piloted version** | **The 29-item FORCE-index** | **N=366** | **N=390** |
| **Preparedness** | | | |  |  |
|  | **Competency** (3 items of the 29-item FORCE-index) | | |  |  |
|  | In relation to the pandemic... | | |  |  |
|  | (Please answer using a scale from 0-10, where 0 corresponds to not at all and 10 corresponds to a very large extent) | | |  |  |
| **1** |  | Have you received sufficient information and training on infection prevention and control and the procedures necessary for carrying out your work tasks safely? | Have you received sufficient information and training on infection prevention and control and the procedures necessary for carrying out your work tasks safely? | 0·946 | 0·840 |
| **2** |  | Have you received sufficient other information and training to be able to carry out your work tasks in a professionally sound manner? | Have you received sufficient other information and training to be able to carry out your work tasks in a professionally sound manner? | 0·942 | 0·817 |
| **3** |  | Have you and your colleagues collectively had sufficient competence to perform your work tasks well? | Have you and your colleagues collectively had sufficient competence to perform your work tasks well? | 0·678 | 0·613 |
|  | **Stress management** (3 items of the 29-item FORCE-index) | | |  |  |
|  | In relation to the pandemic... | | |  |  |
|  | (Answer options: never \| rarely \| sometimes \| often \| nearly all the time, all items are coded 0-10) | | |  |  |
| - |  | Have you received sufficient information about common stress reactions, what might help and where employees can get useful assistance if needed? |  | Single item |  |
| **4** |  |  | Have you received sufficient information about common stress reactions? |  | 0·941 |
| **5** |  |  | Have you received sufficient information about what might help with common stress reactions? |  | 1·004 |
| **6** |  |  | Have you received sufficient information about where employees experiencing stress reactions can get useful assistance? |  | 0·818 |
| **Workday manageability** | | | |  |  |
|  | **Familiarity** (3 items of the 29-item FORCE-index) | | |  |  |
|  | How often during the last month…. | | |  |  |
|  | (Answer options: never \| rarely \| sometimes \| often \| nearly all the time, all items are coded 0-10) | | |  |  |
| **7** |  | Have you known where you would be spending your workday/shift before arriving at work? | Have you known where you would be spending your workday/shift before arriving at work? | 0·631 | 0·585 |
| **8** |  | Have you known who your co-workers would be before arriving at work? | Have you known who your co-workers would be before arriving at work? | 0·861 | 0·889 |
| **9** |  | Have you worked with your regular, familiar colleagues? | Have you worked with your regular, familiar colleagues? | 0·664 | 0·462 |
|  | **Workload manageability** (4 items of the 29-item FORCE-index, explained variance | | |  |  |
|  | How often during the last month…. | | |  |  |
|  | (Answer options: never \| rarely \| sometimes \| often \| nearly all the time, all items have been reversed and are coded 0-10) | | |  |  |
| **10** |  | Have you skipped necessary breaks (eating/going to the bathroom/resting) due to high work pressure? | Have you skipped necessary breaks (eating/going to the bathroom/resting) due to high work pressure? | 0·626 | 0·641 |
| **11** |  | Have you been understaffed due to quarantine/infection/welfare leave, etc., among employees? | Have you been understaffed due to quarantine/infection/welfare leave, etc., among employees? | 0·566 | 0·753 |
| **12** |  | Has your workload been so overwhelming/high that you have lost track and control? | Has your workload been so high that you have lost track and control? | 0·751 | 0·761 |
| **13** |  |  | Have you experienced increased workload due to absence among your colleagues (quarantine/infection/welfare leave, etc.)? |  | 0·700 |
|  | **Work performance** (3 items of the 29-item FORCE-index) | | |  |  |
|  | How often during the last month…. | | |  |  |
|  | (Answer options: never \| rarely \| sometimes \| often \| nearly all the time, all items are coded 0-10) | | |  |  |
| - |  | Have you/your team satisfactorily completed your work tasks by the end of the workday/shift? |  | Single item |  |
| **14** |  |  | Have you/your team had the opportunity to provide the patient(s) you are responsible for with the necessary physical care? |  | 0·724 |
| **15** |  |  | Have you/your team had the opportunity to provide the patient(s) you are responsible for with the necessary psychological care? |  | 0·755 |
| **16** |  |  | Have you/your team had the opportunity to provide the patient(s) you are responsible for with the necessary medical treatment? |  | 0·525 |
| **Hazard protection** | | | |  |  |
|  | **Infection safety** (2 items of the 29-item FORCE-index) | | |  |  |
|  | How often during the last month…· | | |  |  |
|  | (Answer options: never \| rarely \| sometimes \| often \| nearly all the time, all items have been reversed and coded 0-10) | | |  |  |
| **17** |  | Have you feared getting infected? | Have you feared getting infected? | 0·889 | 0·697 |
| **18** |  | Have you feared infecting others (patients, colleagues, family)? | Have you feared infecting others (patients, colleagues, family)? | 0·783 | 0·554 |
|  | **Personal protective equipment (PPE)** (6 items of the 29-item FORCE-index) | | |  |  |
|  | How often during the last month…· | | |  |  |
|  | (Answer options: never \| rarely \| sometimes \| often \| nearly all the time, all items have been reversed and are coded 0-10) | | |  |  |
| **19** |  | Has the use of PPE hindered the exchange of information necessary for solving your work tasks well? | Has the use of PPE hindered the exchange of information necessary for solving your work tasks well? | 0·552 | 0·558 |
| **20** |  | Has the use of PPE led to physical discomfort (e.g., painful chafing, feeling hot/clammy)? | Has the use of PPE led to physical discomfort (e.g., chafing, feeling hot/clammy, or headaches)? | 0·806 | 0·748 |
| **21** |  | Has the use of PPE caused difficulty breathing or that the mask is not tight-fitting? | Has the use of PPE caused difficulty breathing? | 0·655 | 0·636 |
|  |  | Has the use of PPE led to feelings of social isolation and lack of social/collegial support? |  | 0·482 |  |
| **22** |  |  | Has the use of PPE led to feelings of social isolation (loneliness)? |  | 0·654 |
| **23** |  |  | Has the use of PPE hindered social support between colleagues? |  | 0·691 |
| **24** |  | Have you skipped necessary breaks (eating/going to the bathroom/resting) to conserve PPE? | Have you skipped necessary breaks (eating/going to the bathroom/resting) to conserve PPE? | 0·419 | 0·572 |
| **Social environment** | | | |  |  |
|  | **Social safety** (3 items of the 29-item FORCE-index) | | |  |  |
|  | How often during the last month… | | |  |  |
|  | (Answer options: never \| rarely \| sometimes \| often \| nearly all the time, all items have been reversed and are coded 0-10) | | |  |  |
| **25** |  | Have you been blamed, threatened, or excluded by your closest colleagues? | Have you been blamed, threatened, or excluded by your closest colleagues? |  | 0·596 |
| **26** |  | Have you been blamed, threatened, or excluded by your immediate superiors? | Have you been blamed, threatened, or excluded by your immediate superiors? |  | 0·859 |
| **27** |  |  | Have you experienced increased conflict at work due to your own absence (quarantine, illness, welfare leave, etc·)? |  | 0·512 |
|  | **Social support** (2 items of the 29-item FORCE-index) | | |  |  |
|  | How often during the last month…· | | |  |  |
|  | (Answer options: never \| rarely \| sometimes \| often \| nearly all the time, all items are coded 0-10) | | |  |  |
| **28** |  | Have you received support and assistance from your closest colleagues when you needed it? | Have you received support and assistance from your closest colleagues when you needed it? | 0·857 | 0·485 |
| **29** |  | Have you received support and assistance from your immediate superiors when you needed it? | Have you received support and assistance from your immediate superiors when you needed it? | 0·619 | 0·494 |
| Extraction Method: Principal Axis Factoring· Rotation Method: Promax with Kaiser Normalization· T1: Rotation convergence in 9 iterations, T2: Rotation convergence in 8 iterations  PPE; personal protective equipment | | | | | |

| **Supplementary Table 4a.** Total variance explained, T1 (Pilot). | | | | | | | |
| --- | --- | --- | --- | --- | --- | --- | --- |
| **Factor** | Initial Eigenvalues | | | Extraction Sums of Squared Loadings | | | Rotation Sums of Squared Loadings^a^ |
|  | Total | % of Variance | Cumulative % | Total | % of Variance | Cumulative % | Total |
| **1** | 8.538 | 28.460 | 28.460 | 8.138 | 27.126 | 27.126 | 5.486 |
| **2** | 2.684 | 8.947 | 37.408 | 2.293 | 7.643 | 34.769 | 4.405 |
| **3** | 1.984 | 6.615 | 44.023 | 1.505 | 5.015 | 39.784 | 4.329 |
| **4** | 1.575 | 5.250 | 49.273 | 1.202 | 4.008 | 43.792 | 4.177 |
| **5** | 1.452 | 4.840 | 54.113 | 1.025 | 3.416 | 47.208 | 4.716 |
| **6** | 1.257 | 4.191 | 58.304 | 0.830 | 2.766 | 49.974 | 5.157 |
| **7** | 1.025 | 3.417 | 61.721 | 0.626 | 2.085 | 52.059 | 3.769 |
| **8** | 0.939 | 3.130 | 64.851 | 0.473 | 1.576 | 53.636 | 4.563 |
| **9** | 0.878 | 2.927 | 67.778 | 0.317 | 1.058 | 54.694 | 1.498 |

| **Supplementary Table 4b** Total variance explained, T2. | | | | | | | |
| --- | --- | --- | --- | --- | --- | --- | --- |
| Factor | Initial Eigenvalues | | | Extraction Sums of Squared Loadings | | | Rotation Sums of Squared Loadings^a^ |
|  | Total | % of Variance | Cumulative % | Total | % of Variance | Cumulative % | Total |
| 1 | 10.166 | 21.629 | 21.629 | 9.658 | 20.549 | 20.549 | 4.944 |
| 2 | 3.813 | 8.112 | 29.742 | 3.412 | 7.259 | 27.808 | 6.618 |
| 3 | 2.431 | 5.172 | 34.914 | 1.984 | 4.221 | 32.028 | 5.606 |
| 4 | 1.895 | 4.032 | 38.947 | 1.368 | 2.910 | 34.939 | 6.206 |
| 5 | 1.820 | 3.873 | 42.820 | 1.315 | 2.799 | 37.737 | 4.705 |
| 6 | 1.768 | 3.762 | 46.581 | 1.226 | 2.609 | 40.346 | 4.615 |
| 7 | 1.538 | 3.273 | 49.854 | 0.997 | 2.121 | 42.467 | 4.503 |
| 8 | 1.438 | 3.061 | 52.915 | 0.921 | 1.959 | 44.425 | 4.359 |
| 9 | 1.392 | 2.962 | 55.877 | 0.787 | 1.675 | 46.100 | 2.172 |

| **Supplementary Table 5.** Excluded items during preparatory developmental phase of the Frontline health workers’ Occupational Risk and Characteristics in Emergencies index (FORCE-index) | | | |
| --- | --- | --- | --- |
|  |  | **T1 (Pilot)** | **T2** |
|  |  | **10 excluded items at T1** | **24 excluded items at T2** |
| · | | | |
|  | How often during the last month…·(Alternative answers: never \| rarely \| sometimes \| often \| nearly all the time) | | |
| - |  | Has a lack of respirators or other necessary aids prevented you (attending team) from providing the necessary treatment | Has a lack of ventilators or other necessary aids prevented you (treating team) from providing necessary treatment |
| - |  | Have you worn a face mask/respiratory protective device, eye protection/visor, gloves or a protective coat? |  |
| - |  |  | Have you worn simple PPE such as face masks and/or gloves at work? |
| - |  |  | Have you worn advanced PPE such as respiratory protective devices, eye protection/visors, gloves and protective coats? |
| - |  |  | Have you experienced that your mask is not tight? |
| - |  |  | Have infection control measures hindered you from providing patients with necessary follow-up (e.g. outpatient appointments? |
|  | How often during the last month…·(Alternative answers: never \| rarely \| sometimes \| often \| nearly all the time, all items have been reversed and are coded 0-10) | | |
| - |  | Have you worked overtime? | Have you worked overtime? |
| - |  | Have you had to perform tasks you thought could lead to serious errors and that should have been done differently? |  |
| - |  |  | Have you/your team completed your work tasks in a satisfactory manner during the working day/shift? |
| - |  | Have you felt safe at work? | Have you felt safe at work? |
| - |  |  | Have you experienced that others have been afraid of being infected by you? |
| - |  |  | Have you been blamed or threatened by patients or their relatives? |
| - |  |  | Have you found that others have avoided being in contact with your family for fear of being infected? |
| - |  |  | Have you experienced increased workload due to absence among colleagues (quarantine/infection/welfare leave, etc·)? |
| - |  |  | Have you experienced increased workload due to your own absence (quarantine/infection/welfare leave, etc·)? |
| - |  |  | Have you experienced increased conflict in the workplace due to colleagues’ absence (quarantine/infection/welfare leave, etc·)? |
| - |  |  | Have you experienced increased conflict in the workplace due to your own absence (quarantine/infection/welfare leave, etc·)? |
| - |  |  | Have you experienced increased conflict at home due to your job? |
|  |  |  | Have you been afraid to make mistakes that could have serious consequences for your patient(s)? |
|  |  |  | Have you/your team made errors that have had serious medical consequences for the patient(s)? |
|  |  |  | do you experience that you are well protected against infection at work? |
|  | In relation to the pandemic, has the hospital···· | | |
|  | (Please answer using a scale from 0-10, where 0 corresponds to not at all and 10 corresponds to a very large extent) | | |
|  |  | implemented appropriate and professionally sound infection control measures? | implemented professionally sound infection control measures? |
|  |  | organised employees in the best possible manner (with regard to expertise)? | organised employees in the best possible manner (with regard to competency)? |
|  |  | implemented appropriate shift schedules (e.g. adequate rest/recovery)? | implemented appropriate shift schedules (e.g. adequate rest/recovery)? |
|  |  | implemented necessary stress-mitigating measures for employees? | implemented necessary stress-reducing measures for employees? |
|  | During the last month, have you····(Please answer using a scale from 0-10, where 0 corresponds to not at all and 10 corresponds to a very large extent) | | |
|  |  | received the help and support you need at work through measures implemented by the hospital for the purpose of reducing stress? | received the help and support you need at work through measures implemented by the hospital to reduce stress? |
| PPE; personal protective equipment | | | |

| **Supplementary Table 6. Factor loadings for CFA models** | | | | | | |
| --- | --- | --- | --- | --- | --- | --- |
| Second order factor | First order factor | Indicator, FORCE-index item # | T3 First order factor loadings | T4 First order factor loadings | T3 Second order factor loadings | T4 Second order factor loadings |
| Preparedness | | |  |  |  |  |
|  | Competency | | - | - | 0·765 | 0·753 |
|  |  | **1** | 0·816 | 0·773 | 0·816 | 0·773 |
|  |  | **2** | 0·899 | 0·903 | 0·902 | 0·909 |
|  |  | **3** | 0·793 | 0·785 | 0·791 | 0·780 |
|  | Stress management | | - | - | 0·508 | 0·472 |
|  |  | **4** | 0·969 | 0·948 | 0·972 | 0·946 |
|  |  | **5** | 0·967 | 0·943 | 0·966 | 0·943 |
|  |  | **6** | 0·815 | 0·881 | 0·813 | 0·881 |
| Workday manageability | | |  |  |  |  |
|  | Familiarity | | - | - | 0·383 | 0·312 |
|  |  | **7** | 0·471 | 0·590 | 0·461 | 0·594 |
|  |  | **8** | 0·749 | 0·763 | 0·764 | 0·789 |
|  |  | **9** | 0·722 | 0·757 | 0·716 | 0·728 |
|  | Workload manageability | | - | - | 0·742 | 0·652 |
|  |  | **10** | 0·778 | 0·720 | 0·780 | 0·718 |
|  |  | **11** | 0·709 | 0·695 | 0·703 | 0·688 |
|  |  | **12** | 0·729 | 0·764 | 0·737 | 0·777 |
|  |  | **13** | 0·717 | 0·728 | 0·713 | 0·723 |
|  | Work performance | | - | - | 0·188 | 0·173 |
|  |  | **14** | 0·887 | 0·799 | 0·803 | 0·786 |
|  |  | **15** | 0·893 | 0·883 | 1·027 | 0·972 |
|  |  | **16** | 0·572 | 0·648 | 0·441 | 0·517 |
| Hazard protection | | |  |  |  |  |
|  | Infection safety | | - | - | 0·621 | 0·658 |
|  |  | **17** | 0·782 | 0·760 | 0·772 | 0·763 |
|  |  | **18** | 0·870 | 0·797 | 0·881 | 0·793 |
|  | Personal protective equipment (PPE) | | - | - | 0·767 | 0·795 |
|  |  | **19** | 0·651 | 0·480 | 0·650 | 0·475 |
|  |  | **20** | 0·658 | 0·675 | 0·661 | 0·675 |
|  |  | **21** | 0·587 | 0·689 | 0·590 | 0·694 |
|  |  | **22** | 0·701 | 0·658 | 0·707 | 0·667 |
|  |  | **23** | 0·712 | 0·642 | 0·716 | 0·646 |
|  |  | **24** | 0·509 | 0·485 | 0·490 | 0·469 |
| Social environment | | |  |  |  |  |
|  | Social safety | | - | - | 0·544 | 0·458 |
|  |  | **25** | 0·445 | 0·388 | 0·437 | 0·404 |
|  |  | **26** | 0·593 | 0·669 | 0·605 | 0·724 |
|  |  | **27** | 0·594 | 0·588 | 0·586 | 0·551 |
|  | Social support | | - | - | 0·767 | 0·670 |
|  |  | **28** | 0·769 | 0·742 | 0·723 | 0·645 |
|  |  | **29** | 0·946 | 0·911 | 1·006 | 1·048 |

**Frontline health workers’ Occupational Risk and Characteristics in Emergencies index (FORCE-index)**

**OVERVIEW**:

The FORCE index was developed to quantitatively assess frontline workers’ job characteristics and tasks, in responding to an infectious outbreak. The FORCE-index is freely available in the public domain to all interested parties. The structural validity and internal consistency of the instrument has been evaluated in Norwegian. Here, we provide an English translation of the instrument. For translating the tool into other languages, please contact the original authors.

The attached instrument focus on 9 facets of work environment important during pandemics: Competency (item 1-3), Stress management (item 4-6), Familiarity (item 7-9), Workload manageability (item 10-13), Work performance (item 14-16), Infection Safety (item 17-18), Personal Protective Equipment (item 19-24), Social safety (25-27), and Social support (item 28-29).

The development of the scale is described in detail in Stensland et al., Public Health in Practice, 2025, <https://doi.org/10.1016/j.puhip.2025.100582>.

**SCORING RULES** are as follows:

- Items 1 to 6 are scored 1-10.

- Remaining items (7-29) are scored on a five points scale, using the following anchors:

Never, Rarely, Sometimes, Often, Almost always.

For scoring, Items 1-9, 14-16, and 28-29 anchors are transformed into scores 0, 2.5, 5, 7.5, 10.

Remaining Items 10-13, 17-27 anchors are transformed into reversed scores 10, 7.5, 5, 2.5, 0.

Subscale scores are calculated according to the below:

Competency = mean of items 1-3,

Stress management = mean of items 4-6,

Familiarity = mean of items 7-9,

Workload manageability = mean of reversed items 10-13,

Work performance = mean of items 14-16,

Infection Safety = mean of reversed items 17-18,

Personal Protective Equipment = mean of reversed items 19-24,

Social safety = mean of reversed items 25-27,

Social support= mean of items 28-29.

Higher subscale scores indicate a higher assessment of work environment in that specific facet.

**THE REFERENCE** for the measure is: S.Ø. Stensland, K. Bondjers, J.A. Zwart, L.A. Rosseland, D. Atar, J.O. Christensen, D. Matre, K.A. Glad, T. Wentzel-Larsen, H. Wøien, G. Dyb. Development and psychometric validation of the Frontline health workers’ Occupational Risk and Characteristics in Emergencies index (FORCE-index) – The Covid Hospital Cohort Study, Public Health in Practice, 2025, 100582, ISSN 2666-5352, <https://doi.org/10.1016/j.puhip.2025.100582>. (https://www.sciencedirect.com/science/article/pii/S2666535225000011)

**Frontline health workers’ Occupational Risk and Characteristics in Emergencies index (FORCE-index)**

In connection with the pandemic …

(Answer on a scale from 0–10, where 0 corresponds to “not at all” and 10 corresponds to “to a large degree”)

|  |  | Not at all | |  |  |  |  |  |  | To a large degree | | |
| --- | --- | --- | --- | --- | --- | --- | --- | --- | --- | --- | --- | --- |
| 1 | have you received sufficient information and training on infection prevention and control and the procedures necessary for carrying out your work tasks safely? | 0 | 1 | 2 | 3 | 4 | 5 | 6 | 7 | 8 | 9 | 10 |
| 2 | have you received sufficient other information and training to be able to carry out your work tasks in a professionally sound manner? | 0 | 1 | 2 | 3 | 4 | 5 | 6 | 7 | 8 | 9 | 10 |
| 3 | have you and your colleagues collectively had sufficient competence to perform your work tasks well? | 0 | 1 | 2 | 3 | 4 | 5 | 6 | 7 | 8 | 9 | 10 |
| 4 | have you received sufficient information about common stress reactions? | 0 | 1 | 2 | 3 | 4 | 5 | 6 | 7 | 8 | 9 | 10 |
| 5 | have you received sufficient information about what might help with common stress reactions? | 0 | 1 | 2 | 3 | 4 | 5 | 6 | 7 | 8 | 9 | 10 |
| 6 | have you received sufficient information about where employees experiencing stress reactions can get useful assistance? | 0 | 1 | 2 | 3 | 4 | 5 | 6 | 7 | 8 | 9 | 10 |

How often during the last month …

(Answer ticking the appropriate box below)

|  |  | Never | Rarely | Some-times | Often | Almost always |
| --- | --- | --- | --- | --- | --- | --- |
| 7 | have you known where you would be spending your workday/shift before arriving at work? |  |  |  |  |  |
| 8 | have you known who your co-workers would be before arriving at work? |  |  |  |  |  |
| 9 | have you worked with your regular, familiar colleagues? |  |  |  |  |  |
| 10 | have you skipped necessary breaks (eating/going to the bathroom/resting) due to high work pressure? |  |  |  |  |  |
| 11 | have you been understaffed due to quarantine/infection/welfare leave, etc., among employees? |  |  |  |  |  |
| 12 | has your workload been so high that you have lost track and control? |  |  |  |  |  |
| 13 | have you experienced increased workload due to absence among your colleagues (quarantine/infection/welfare leave, etc.)? |  |  |  |  |  |
| 14 | have you/your team had the opportunity to provide the patient(s) you are responsible for with the necessary physical care? |  |  |  |  |  |
| 15 | have you/your team had the opportunity to provide the patient(s) you are responsible for with the necessary psychological care? |  |  |  |  |  |
| 16 | have you/your team had the opportunity to provide the patient(s) you are responsible for with the necessary medical treatment? |  |  |  |  |  |
| 17 | have you feared getting infected? |  |  |  |  |  |
| 18 | have you feared infecting others (patients, colleagues, family)? |  |  |  |  |  |
| 19 | has the use of personal protective equipment hindered the exchange of information necessary for solving your work tasks well? |  |  |  |  |  |
| 20 | has the use of personal protective equipment led to physical discomfort (e.g., chafing, feeling hot/clammy, or headaches)? |  |  |  |  |  |
| 21 | has the use of personal protective equipment caused difficulty breathing? |  |  |  |  |  |
| 22 | has the use of personal protective equipment led to feelings of social isolation (loneliness)? |  |  |  |  |  |
| 23 | has the use of personal protective equipment hindered social support between colleagues? |  |  |  |  |  |
| 24 | have you skipped necessary breaks (eating/going to the bathroom/resting) to conserve personal protective equipment? |  |  |  |  |  |
| 25 | have you been blamed, threatened, or excluded by your closest colleagues? |  |  |  |  |  |
| 26 | have you been blamed, threatened, or excluded by your immediate superiors? |  |  |  |  |  |
| 27 | have you experienced increased conflict at work due to your own absence (quarantine, illness, welfare leave, etc.)? |  |  |  |  |  |
| 28 | have you received support and assistance from your closest colleagues when you needed it? |  |  |  |  |  |
| 29 | Have you received support and assistance from your immediate superiors when you needed it? |  |  |  |  |  |
